# Supplementary material for: Ecological Drivers of Community Cohesion
Source: mSystems. 2023 Jan 19;8(1):e00929-22. doi: 10.1128/msystems.00929-22 (PMC9948702; doi:10.1128/msystems.00929-22)
Supplement: TEXT S1 [file msystems.00929-22-s0008.pdf]

Ecological Drivers of Community Cohesion - Supplementary Materials

Supplementary Materials & Methods

Saccharomyces cerevisiae Strains. Oligo primers used in this study are listed in `https://github.com/tecoevo/ecoblocs/blob/main/strain_creation/primer_list.csv` and produced by Integrated DNA Technologies (Singapore). Strain development relied on creation of a progenitor strain, from w303-1, with auxotrophies for adenine, lysine, tryptophan and histidine and the insertion of feed back resistant (FBR) mutations that result in the over production of either lysine, tryptophan or histidine. Specifically, the KanMX, derived from plasmid p0003 (Addgene 44901), marker was inserted into the *HML*α loci using primers P0114 and P0115 to prevent mating type switching. *ADE4* was disrupted with *CaURA3* amplified from plasmid p0011 using primers P0118 and P0119. *LYS2*+ was disrupted using function copy of *LEU2*+ amplified from W303-2 genomic DNA using primers P0123 and P0124. This resulted in the Progenitor strain.

The *ADE*↑ strain was created by replacing *ade4::CaURA5* with *ADE4(PUR6)*, the FBR version of *ADE4*, amplified from yeast strain WS950 using primers P0155 and P0156. *URA3*+ amplified from WS950 using primers P0069 and P0070. The *HIS*↑ strain was created by insertion of (*HIS1FBR, HIS3*) from plasmid p0050 into the *HIS1* locus using primers P0151 and P0152. The *TRP*↑ strain was created by insertion of (*TRP2FBR, TRP1*), from plasmid p0044, using primers P0309 and P0310. The *LYS*↑ strain was created by insertion of (*LYS21FBR, LYS2*), from plasmid p0045, using primers P0312 and P0313.

Plasmids. A list of plasmids used in this study is provided in `https://github.com/tecoevo/ecoblocs/blob/main/strain_creation/plasmid_list.csv`. Gibson assembly was used for plasmid creation (E2611L - New England Biolabs(NEB), Tokyo, Japan). Plasmids p0003 and p0011 were ordered from AddGene (Watertown, MA, USA) (plasmid IDs 44901 and 44650 respectively). The plasmid pUC19 (N3041S- NEB) was used as a backbone for all plasmids constructed. Plasmid p0050 (*HIS*↑) was created by cloning *HIS1* and *HIS3* using primers P0145-P0148 into pUC19. *HIS1* was mutated to the feedback resistant form described in [1] using primers P0149 and P0150. Plasmid p0044 (*TRP*↑) was created by cloning *TRP2FBR* and *TRP1* using primers P0157-P0160. *TRP2FBR* was amplified

from yeast strain yMM65 [2]. Plasmid p0045 (*LYS*↑) was created by cloning *LYS21FBR* and *LYS2* using primers P0161-P0164 into pUC19. *LYS21FBR* was amplified from yeast strain WS954 using primers P0161 and P0162.

Culturing. All cultures were grown at $30\,^{\circ}\mathrm{C}$ in an orbital shaker at 200RPM. Each experiment was performed in triplicate starting from a single individual colony. Experimental cultures were generated by selecting individual colonies from a streak plate and growing each colony in $5\,\mathrm{mL}$ SC medium for 48 hours. These cultures were pelleted, washed twice with SC-aa and resuspended in $5\,\mathrm{mL}$ of SC-aa. They were then grown for an additional 24 hours. This allowed the culture to reach a carrying capacity of approximately 5×10^7. These cultures were pelleted, washed and resuspended as above and then were diluted 1 in 20 with SC-aa. Thus, all experiments were at approximately one twentieth of the carrying capacity. Experimental cultures were generated by mixing individual strain dilutions in equal proportions to a final volume of $5\,\mathrm{mL}$. Nutrients were added as needed to a final concentration of 100 µg/mL at timepoint 0. The cultures were sampled to determine growth and strain ratios every 24 hours (including timepoint 0). All experiments were performed in triplicate.

Cell counts, Strain Ratios & Culture Growth. All experimental cultures were sampled by removing 20µl every 24 hours. Cell counts per millilitre of viable cells were obtained from experimental cultures using the Muse cell counter (Merck Millipore) with the Cell Count & Viability kit per the manufacturers instructions. To obtain strain ratios, samples were serially diluted, played on YPD solid media, allowed to grow for two days and colonies were counted. These plates were then replicated onto solid media that permitted growth of only one strain, SC without lysine, adenine, tryptophan or histidine, counts of each strain were obtained, and ratio calculated. Growth rates were calculated for all pair-wise strain combinations as well as all four strains together. Growth rate was determined as the average growth across the first three days of growth.

Estimating growth parameters. We determined growth rates of each strain in our four-strain system when either grown together, four strains per culture, and in all pairwise combinations, two strains per culture. To reduce temporal-induced variation, growth values are the average of three days growth. Growth rate was calculated using the formula "(cells at time $t+1$ - cells at time t) / cells at time t" for each replicate or each culture. For determining pool-growth we used the 4-strain cultures with growth averaged over three days. This was *ADE*↑ :1.028 / *TRP*↑ :0.282 / *HIS*↑ :1.564 and *LYS*↑ :1.103 (Fig. SI.1). Triple-wise and varying ecology strain growth rates were estimated by averaging growth rates of each relevant pair-wise and four-strain growth rates. For example, to calculate the *ADE*↑ strain growth rate when grown with *HIS*↑ and *TRP*↑ , the growth rate is the average of the four-strain growth rate and the growth rates from the *ADE*↑ / *HIS*↑ and *ADE*↑ / *TRP*↑ pair-wise growth rates. Synthetic

mutualism was determined through growth in conditions that required four-way cross-feeding. We calculated growth rates in all pairwise combinations of the engineered strains and with the four pooled strains. From these experiments, we extrapolate growth rates of the individual strains when present in different community compositions and when in a variety of ecologies. These growth rates are shown in Fig. SI.2. Furthermore, an alternative method for calculation of growth rates has been included in Fig. SI.3. In addition raw data and growth rates are provided on (GitHub).

Alternative estimation of growth parameters. Estimating the growth rates of individual strains given the community and the ecology is non-trivial. Due to the lack of experimental data we hypothesize the growth rate from our estimates from a subset of experiments. Since the extrapolation can be done in several ways here we discuss another method and display the results accordingly. We identified no key differences between these methods in terms of the conclusions drawn from this work. This suggested that regardless of how this interpolation was achieved, the influence was negligible as it was still tightly bound by the experimental data. The alternative method, more subjective method, employed a maximum growth strategy (Fig. SI.3). Herein the highest growth rates of each strain were favored in media with a higher degree of supplementation. Where all required nutrients were provided this highest rate was used. In conditions requiring inter-strain cross feeding was required, growth rates from pairwise interactions were employed. However, this did serve to increase overall differences between the strongest strain, *HIS*↑ , and the weakest strain, *TRP*↑ . The data is available at `https://github.com/tecoevo/ecoblocs/tree/main/growth_data`

Supplementary Results & Discussion

The need for ecological structure

A basic assumption of our system is the cycling processes between a structured ecology and a well-mixed pool. Therefore as a null model, we contrast it with the case where no structure exists - hence a single well. We start the single well with a random initial condition and an initial environmental setting. We present the mean of 100 such runs in Fig. SI.5. We observe that almost all lineages go extinct irrespective of the time spent in the pool phase (concerning $t_{well} = 20$). If we include metabolic feedback, the strains change their environment negatively (Fig. 5 main text). For a single well, there is no escape possible as offered by the population structure. Hence, all lineages go extinct Fig. SI.5 (bottom). This exercise demonstrates the inadequacy of a single well to survive the cycling environmental regime.

Evolutionary fate

We have previously discussed a two strain synthetic system [3] based on the works of [4]. The engineered strains lacked the production of adenine or lysine. A code could define the strains: $\{m_1, m_2\}$ where the production of a metabolite (m) can be represented by on 1 or off 0. Thus the wild-type is denoted as $\{1, 1\}$ and the two constructed strains are $\{1, 0\}$ and $\{0, 1\}$. The latter two can grow only in a rich environment where the unsecreted metabolite is present or in the presence of each other where they support each other via the engineered mutualism. However, binary systems are typically exceptional cases of complex systems [5]. We augmented the two-strain systems with different interdependencies, now tryptophan and histidine coming from [2]. Engineering different levels of interdependencies with four metabolites can be represented by $\{m_1, m_2, m_3, m_4\}$ where again we have $m_i = \{0, 1\}$. Thus 16 different strains are possible. Our study focused on the extreme four, where the strain can produce only one metabolite.

The costs and benefits of acquiring and losing essential genes can be highly complicated and environment-dependent [6, 7]. According to the Black Queen hypothesis, the loss of essential genes for leaky products can be feasible if the environment can compensate. Furthermore, gaining new genes might not always come at a growth cost. A thorough analysis of the cost-benefit structure in the light of the ecology is, therefore, necessary for systems that can incorporate the evolution of these interdependence traits [8, 9]. The possibility of tuning the strengths of interspecific interactions can, in turn, also affect the observed biodiversity levels in evolving communities [10].

Keystone

If the environment cannot provide what is essential for survival then the organism that can becomes a keystone of a community that thrives there. We show this intuition by removing all environments that involve tryptophan. *TRP*↑ is the slowest growing strain but now it becomes important as it is the only source of TRP for the rest of the strains. As seen in Figure SI.7 , *TRP*↑ can grow to appreciable frequencies and even replace the next slower growing strain *ADE*↑ .

References

[1] Borstel, R. C. v. *et al.* Topical Reversion at the HIS1 Locus of Saccharomyces cerevisiae * A Tale of Three Mutants. *Genetics* **148**, 1647–1654 (1998).

[2] Müller, M. J. I., Neugeboren, B. I., Nelson, D. R. & Murray, A. W. Genetic drift opposes

mutualism during spatial population expansion. *Proceedings of the National Academy of Sciences of the United States of America* **111**, 1037–1042 (2014).

[3] Denton, J. A. & Gokhale, C. S. Promoting Synthetic Symbiosis under Environmental Disturbances. *mSystems* **5**, e00187–20 (2020).

[4] Shou, W., Ram, S. & Vilar, J. M. G. Synthetic cooperation in engineered yeast populations. *Proceedings of the National Academy of Sciences of the United States of America* **104**, 1877–1882 (2007).

[5] Gokhale, C. S. & Traulsen, A. Evolutionary games in the multiverse. *Proceedings of the National Academy of Sciences USA* **107**, 5500–5504 (2010).

[6] Morris, J. J. Black Queen evolution: the role of leakiness in structuring microbial communities. *Trends in Genetics* **31**, 475–482 (2015).

[7] Kafri, M., Metzl-Raz, E., Jona, G. & Barkai, N. The Cost of Protein Production. *Cell Reports* **14**, 22–31 (2016).

[8] Zomorrodi, A. R. & Segrè, D. Genome-driven evolutionary game theory helps understand the rise of metabolic interdependencies in microbial communities. *Nature Communications* **8**, 6449 (2017).

[9] Oña, L. *et al.* Obligate cross-feeding expands the metabolic niche of bacteria. *Nature Ecology & Evolution* 1–9 (2021).

[10] Rivett, D. W. *et al.* Resource-dependent attenuation of species interactions during bacterial succession. *The ISME journal* (2016).
